# Increased urge for movement, physical and mental restlessness, fundamental symptoms of restricting anorexia nervosa?

**DOI:** 10.1002/brb3.1556

**Published:** 2020-02-04

**Authors:** Regina C. Casper, Ulrich Voderholzer, Silke Naab, Sandra Schlegl

**Affiliations:** ^1^ Department of Psychiatry and Behavioral Sciences Stanford University School of Medicine Stanford CA USA; ^2^ Schön Klinik Roseneck Prien am Chiemsee Germany; ^3^ Department of Psychiatry and Psychotherapy University Hospital of Munich (LMU) Munich Germany

**Keywords:** anorexia nervosa, drive for activity, restless activation, urge for movement

## Abstract

**Objective:**

Continued mobility in the presence of severe weight loss is a well known, yet insufficiently researched characteristic of anorexia nervosa (AN). This study was designed to assess the prevalence of the drive for activity, here operationalized as an increased urge for movement, physical restlessness, and mental restlessness.

**Method:**

Participants were 83 female consecutively admitted adolescent patients qualifying for a diagnosis of AN (ICD‐10), restricting subtype. Information collected included responses to a questionnaire inquiring retrospectively about physical and psychological reactions after significant weight loss (on average 12.5 kg) and to measures of psychiatric and eating disorder pathology and exercise behaviors at hospital admission.

**Results:**

Over 80% of AN patients reported experiencing, at least partly, either, an increased urge for movement, physical or mental restlessness after significant weight loss. Altogether 95.1% reported, at least partly, one or a combination of two or all three symptoms. The sensations coexisted with equally high levels of fatigue and loss of energy, typically observed in starvation. The increased urge for movement and physical restlessness were foremost associated with reported actual physical activity and with weight loss. By contrast, mental restlessness was strongly linked to the degree of eating disorder pathology and to the severity of psychiatric symptoms.

**Discussion:**

This is the first investigation of the presence of an increased urge for movement, physical restlessness, and mental restlessness after significant weight loss in patients with acute AN. The symptoms, given their high frequency and specificity, are likely pathogenic for AN and, if replicated, deserve to be considered for inclusion as diagnostic criteria for AN.

## Graphical abstract

Continued mobility in the presence of severe weight loss is a well‐known, yet insufficiently researched characteristic of anorexia nervosa (AN). This study explored the question whether and to what extent AN patients might be aware of an increased urge to move and physical and mental‐restlessness at the time of their greatest weight loss. Over 80% of AN patients reported experiencing either, an increased urge for movement, physical or mental restlessness after significant weight loss. Altogether 95.1% reported one or a combination of two or all three symptoms. These findings suggest a unique physiological adaptation of energy regulating pathways in AN.

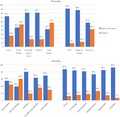

## 1 INTRODUCTION

Anorexia nervosa (AN) is a serious disorder of voluntary food restriction primarily occurring in female adolescents leading to excessive weight loss. Recent evidence suggests that AN is a complex heritable phenotype (Watson et al., 2019). Prolonged severe energy restriction in humans is generally accompanied by a host of physiological and metabolic adaptations. These adjustments, designed to conserve energy, eventually lead to a reduction in and avoidance of physical activity, inertia, and apathy (Keys, Brozek, Henschel, Mickelsen, & Taylor, 1950). The early literature reveals that clinicians regularly noticed a desire and ability for movement inconsistent with the degree of undernutrition and underweight in patients with anorexia nervosa (Gull, 1874; Lasègue, 1873). Davis et al. (1997) began to systematically interview patients and to document greater than average activity levels in childhood as well as excessive exercise in AN patients in the acute phase of the disease and proposed a central role for physical activity in the development of AN. More recently, a review of studies that measured daily activity using movement sensors and total energy expenditure found that day to day physical activity levels and daily energy expenditure in AN patients did not significantly differ from those in healthy controls (Casper, 2018).

Since, with the exception of nurses' ratings of activity levels on the short anorexic behavior scale (Slade, 1973), eating disorder assessment instruments have not included questions on motor activity, very little is known whether and to what extent AN patients are aware of their continued movements or an urge for movement or physical restlessness, all of which may contribute to the phenomenon of persistent motility in AN.

Holtkamp et al. (2004) asked adolescent AN patients on hospital admission to report the amount of physical activity in the past 3 months. Activity levels were higher with greater food restriction and with greater anxiety. In a subsequent study (Holtkamp et al., 2006) of adolescent AN patients, an inverse relationship with leptin levels was observed between subjective and objective measures of physical activity and motor and inner restlessness. Sternheim, Danner, Adan, and van Elburg (2015) reported positive associations between a “drive for activity” measure and levels of eating disorder pathology and anxiety in adolescent and adult AN patients. Keyes et al. (2015) compared daily movements and “drive to exercise” ratings in adult in‐and outpatients with AN to age‐matched healthy controls and a group diagnosed with anxiety disorders. Actigraph recordings showed no differences between the three groups, but AN patients reported a greater “drive to exercise.” Collectively, these studies reveal an association between the degree of food restriction and the recorded physical activity and restlessness in AN, without reaching agreement concerning the relationship to eating disorder and psychiatric pathology.

To our knowledge, the question, whether and to what extent AN patients might be aware of an increased desire to move, or physical, or mental restlessness, has not been specifically investigated. This study, therefore, was designed (a) to obtain information on the presence/absence and frequency of an increased urge to move and/or physical and/or mental restlessness around the time of the first maximum weight loss. Given the patients' tendency to neglect physical discomfort resulting from undernutrition and underweight, we thought it would be valuable (b) to obtain information about the most commonly occurring physical and psychological reactions recorded in simple starvation (Keys et al., 1950). Another purpose of the study was (c) to explore the relationships between the retrospectively reported increased urge for movement, and physical and mental restlessness, with other parameters of the disease, the eating disorder pathology and psychiatric morbidity at hospital admission.

## 2 MATERIAL AND METHODS

The study included 83 female teenage patients, who were consecutively admitted to a hospital specializing in the Treatment of Eating Disorders from the autumn of 2017 to the spring of 2019. Patients met ICD‐10 criteria for AN (F50.0) (ICD, 2010) and all qualified for a diagnosis of anorexia nervosa (AN), restricting subtype, using a standardized interview. Patients were excluded from the study, if they met criteria for a diagnosed medical illness or another Axis I disorder, except for anxiety disorders or major depressive disorders.

Data collection protocols were submitted to and approved by the Ethics Committee of the Faculty of Medicine at Ludwig Maximilian University (LMU) Munich, Germany. fInformed written consent was provided by all participants, including their parents, for patients younger than 18 years.

### 2.1 Measures

Demographic information included age, BMI [weight (kg)/height (m^2^)], amount of weight loss, and duration of illness on admission. To stay close to the onset of illness and for reasons of homogeneity of diagnosis, teenage patients who fulfilled criteria for acute AN, restricting type, were selected. The responses to the following self‐report questionnaires were collected from all participants.

#### 2.1.1 Reactions to Weight Loss Questionnaire

The form invited patients with acute AN to describe retrospectively their subjective feelings and sensations at the time of their first lowest weight and to report their weight and height, the degree of weight loss and the time to the weight loss. The form was designed to establish the presence or absence and degree of each item in a three‐pronged answer (applies—applies in part—does not apply; Appendix S1). Each patient was asked to endorse or reject eight possible physical and eleven possible psychological reactions following weight loss. The three principal variables (an increased urge for movement—physical restlessness—mental restlessness) were embedded with other sensations such as feeling active, full of energy, motivated, and variables describing feeling states commonly associated with starvation, such as tired, loss of energy, loss of motivation. We did not define the three major variables by intent, in the expectation that the definitions might emerge from the data collected in the study. Patients also indicated whether they had been more active in childhood compared with other children and how well they remembered the information given on the Reactions to Weight Loss Questionnaire (RWLQ). Patients completed the questionnaire during the week following admission to the hospital.

To test whether teenage patients understood and were able to answer the statements, the RWLQ form was first administered to 20 female adolescents diagnosed with AN using ICD‐10 criteria (F50.0), who were hospitalized at the Department of Child and Adolescent Psychiatry, Charité‐Universitätsmedizin Berlin, Germany. All patients completed every item on the questionnaire without difficulty.

#### 2.1.2 Eating Disorder Inventory‐2

The eating disorder inventory‐2 (EDI‐2; Garner, 1983) is used for the multidimensional assessment of the specific psychopathology of patients with eating disorders. It consists of 11 scales with 91 items that can be answered on a six‐point scale from 1 (never) to 6 (always). Cronbach's *α* for the EDI‐2 total sum score for this sample was .962.

#### 2.1.3 Beck Depression Inventory‐II

The Beck depression inventory (BDI‐II; Beck, Steer, & Brown, 1996) is a 21 item self‐report inventory for measuring the severity of depressive symptoms. Items are rated on a four‐point scale from 0 to 3 in terms of their occurrence and intensity during the last 2 weeks. Cronbach's *α* for the BDI‐2 sum total score for this sample was .907.

#### 2.1.4 Brief Symptom Inventory‐18

The brief symptom inventory‐18 (BSI; Franke et al., 2011), a self‐report symptom inventory, assesses the level of current general psychological distress of patients throughout the last week on the basis of 53 items belonging to nine scales: somatization, obsessive‐compulsive, interpersonal sensitivity, depression, anxiety, hostility, phobic anxiety, paranoid ideation, and the general severity index. Each item is rated on a five‐point scale of distress from 0 (not at all) to 4 (extremely) during the last week.

#### 2.1.5 Compulsive exercise test

The compulsive exercise test (CET; Taranis, Touyz, & Meyer, 2011) is a multidimensional measure designed to assess core factors operating in the maintenance of CE specifically among patients with eating disorders. It comprises 24 items and the following 5 subscales: Avoidance and rule‐driven behavior, weight control exercise, mood improvement, lack of exercise enjoyment, and exercise rigidity summed up to a CET total score. Ratings are based on a Likert scale ranging from 0 (never true) to 5 (always true). Cronbach's *α* for the CET total score for this sample was .945.

#### 2.1.6 Commitment to Exercise Scale

The Commitment to Exercise Scale (CES; Davis, Brewer, & Ratusny, 1993) The CES is an eight‐item self‐rating scale frequently used for the assessment of compulsive exercise in patients with eating disorders. In accordance with (Thome & Espelage, 2007), we used a 4‐point Likert scale. It addresses two core aspects of compulsive exercise: obligatory exercising and pathological exercising. Obligatory exercise implies the strict adherence to a regular and clearly structured exercise routine. Pathological exercise refers to the physical or psychological burden caused by the exercise.

#### 2.1.7 Questions regarding your physical activity (rated from 1 to 5)

- Please estimate on the following scale how often you were physically restless in the past 2 weeks, that is, how often did you struggle to sit still without, for example, moving your arms, fidgeting with your legs or tensing your abdominal or leg muscles?
- On the following scale we want you to check how often you organized your everyday life physically more active in the past 2 weeks, that is, how often have you consciously ensured to, for example, bike to school or work instead of taking the bus, to take the stairs instead of the elevator, to carry out strenuous housework, or to take long walks independent of the weather.
- On the last scale we want you to estimate, how often in the past 2 weeks have you consciously ensured to, for example, regularly run, bike, skate, swim, or perform strenuous exercises such as sit‐ups or push‐ups.

### 2.2 Statistical analysis

The analyses of the data from the present study were predominantly descriptive and should be considered as exploratory. Data are presented in relative frequencies (nominal data) and in means and standard deviations for interval scale data. Correlation analyses were done to explore associations between the RWLQ items and other relevant variables. The software used was IBM, SPSS 25.

## 3 RESULTS

The mean age of the AN patients was 16.4 ± 1.9; 84.3% were adolescents from 13 to 18 years old and 15.7% were aged between 19 and 20 years. The BMI was 16.1 ± 1.95 on admission. At the time of the retrospective assessment, the mean minimum BMI was 13.8 ± 1.6, the mean weight in percentile was 5.9 ± 8.9, with an average amount of weight loss of 12.5 ± 5.8 kg within a period of 5.9 ± 4.9 months. Duration of illness was 2.16 ± 1.80 years. Quality of recall was (very) good in 57.3% of patients and (very) weak in 42.7% of the sample.

Sixty‐two percent of patients recalled being more physically active in childhood (true/partly true) than other children, while 38% stated their activity level did not differ from other children.

### 3.1 Physical reactions to weight loss and their frequency

**Figure 1:**
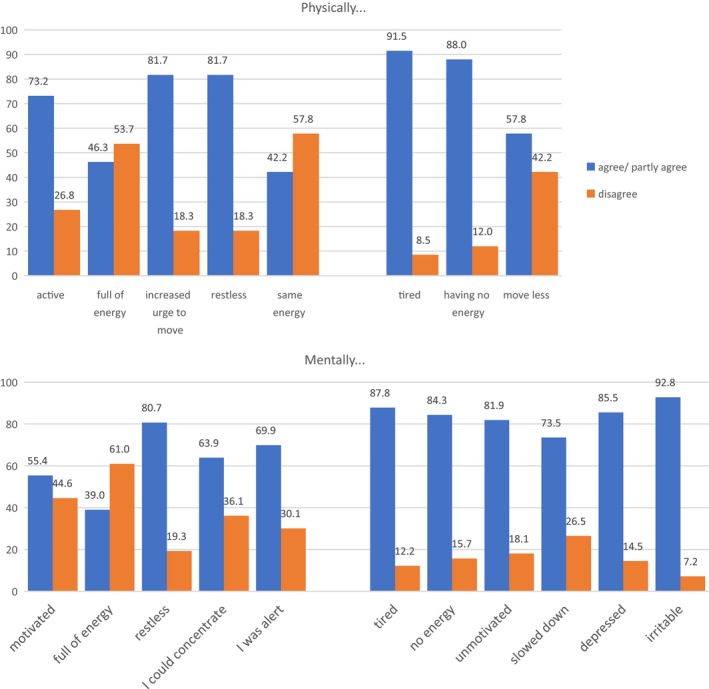
Physical and psychological reactions to the first severe weight loss (on average 12.5 kg) in female teenage anorexia nervosa patients with the restricting subtype (*N* = 83)

The majority (81.7%) of adolescent patients, all of whom qualified for the restricting form of AN, reported experiencing an increased desire or urge for movement after undergoing significant weight loss (agree: 45.1%), (Figure 1). About 81.7% experienced physical restlessness (agree: 42.7%). About 73% reported feeling active. Energy levels differed widely, 46% felt full of energy, in 42% energy levels were unchanged, and 88% endorsed having no energy. These sensations coexisted with high levels (90%) of tiredness, while 58% of patients endorsed a lesser urge to move.

### 3.2 Psychological reactions to weight loss and their frequency

Mental restlessness was reported by over 80% of patients; 70% reported feeling alert, 64% were able to concentrate, and 55% reported feeling motivated with 39% endorsing mental energy. Yet, between 70% and 87% reported feeling at the same time mentally tired, unmotivated, slowed down, or having less mental energy.

### 3.3 Correlational analyses between the increased urge for movement, physical and mental restlessness and all other variables on the RWLQ

We then explored the relationships between the three principal variables and responses to all other items on the RWLQ (Table 1). An increased urge for movement was positively associated with weight loss in percentile and with feeling physically active; it was negatively correlated with a reduced urge to move. There were weak positive associations with being physically restless and mentally restless. Physical restlessness was found to be positively correlated with BMI at weight loss and weight loss in kg. It was highly correlated with mental restlessness, less strongly with mental tiredness as well as with feeling depressed and feeling irritable. Mental restlessness in addition to its high correlation with physical restlessness, showed relationships to physical and mental tiredness, with mental low energy, with feeling depressed and irritable and negatively, with being able to concentrate. Moderate associations were seen to weight loss as BMI and weight loss in kg.

**Table 1:** Significant correlations between the increased urge for movement, physical restlessness, mental restlessness, and all other variables on the Reactions to Weight Loss Questionnaire (RWLQ) as well as minimum BMI, minimum weight in kg, and percentile weight loss

Variable	I felt—an increased urge to move around	Mentally restless	Physically restless
Physically			
I felt—an increased desire to move around		.279*	.284*
Active	.337**		
Tired		.273*	
I preferred to move less	−.374**		
Restless	.284*	.718**	
Mentally			
Tired		.412**	.262*
No energy		.237*	
Restless	.279*		.718**
I could concentrate		−.249*	
Depressed		.442**	.338**
Irritable		.236*	.358**
Weight loss calculated as percentile	.274**		
Weight loss, BMI		.252*	.300**
Weight loss in kg		.261*	.318**

* ≤.05, **≤.01.

### 3.4 The frequency of the occurrence or cooccurrence of an increased urge for movement, physical restlessness and mental restlessness

About 63% of patients endorsed experiencing all three symptoms: an increased urge for movement, physical restlessness, and mental restlessness. An increased urge for movement was present alone or in combination with either form of restlessness in 81.4%. Physical and mental restlessness, only, were reported by 12.3%, while 1.2% reported physical restlessness alone. No patient reported mental restlessness as the only symptom. A mere 4.9% of patients reported experiencing none of the three symptoms.

With regard to excessive activity in childhood, 62% recalled being more active in childhood than other children. The more active group did not differ from the 38% normally active in the frequencies of any of the three principal symptoms.

### 3.5 Symptom severity scores for the eating disorder inventory, the Beck depression inventory, the brief psychiatric symptom inventory and the exercise scales on hospital admission

The EDI‐2 scores for the teenage patients with acute AN (Table 2) reflect moderate to severe eating disorder pathology. The mean and range of the BDI‐II scores suggest the presence of clinically significant depressive symptoms. The levels of psychopathology and psychological distress as reflected in the global severity index of the BSI were in the low to medium range. The CET and CES scores indicated a low to high level of compulsion and commitment to exercise. Similarly, physical restlessness and exercise intensity during the last 2 weeks before admission ranged from low to high, while actual physical activity was in the low to moderate range.

**Table 2:** Mean and standard deviations for the eating disorder, psychiatric symptom, and exercise variables assessed on admission to hospital

Total scores	Mean	Standard deviation
Eating Disorder Inventory‐2 total score	301.83	57.44
Beck Depression Inventory‐2 total score	28.45	12.92
Brief Symptom Inventory Global Severity Index	1.32	0.67
Compulsive exercise test total score	2.64	1.11
Commitment to Exercise Scale total score	2.30	0.84
Physically restless in the last 2 weeks (RWLQ)	2.54	1.56
Daily routine exercise intensity last 2 weeks (RWLQ)	2.52	1.63
Physical activity in the last 2 weeks (RWLQ)	1.35	1.53

### 3.6 Relationships between the retrospectively reported increased urge for movement, physical restlessness and mental restlessness and ratings (summarizing the past 2 weeks before hospital admission) indicating eating disorder pathology, psychiatric symptoms, and commitment to and compulsion for exercise

The increased urge for movement correlated mainly with physical activity and exercise (Table 3) and was significantly linked to daily routine exercise intensity, to continuing to exercise, despite feeling tired or unwell, and with compulsion and commitment to exercise. There were weak correlations to perfectionism on the EDI‐2 and to anxiety on the BSI. Physical restlessness was strongly associated with actual physical restlessness and with agitation rated on the BDI‐II. Other strong correlations were with anxiety on the BSI and with compulsive exercise on the CET. Weaker associations were with interoceptive awareness on the EDI‐2 and commitment to exercise. Mental restlessness showed widespread and strong correlations with most measures on the EDI‐2—the exception were drive for thinness and bulimia—with ineffectiveness, interoceptive awareness, maturity fears, asceticism, impulse regulation, social insecurity, and on the BSI with obsessive‐compulsiveness, interpersonal sensitivity, anxiety, and psychoticism, besides being related to physical restlessness and continuing to exercise despite feeling tired on the CES. On the CET scale rule‐driven behavior, lack of exercise enjoyment, and lack of mood improvement were linked to mental restlessness. Importantly, total scores for the EDI, BSI, CET, and CES were strongly associated with mental restlessness.

**Table 3:** Correlations between the retrospectively reported increased urge for movement, physical restlessness, and mental restlessness with ratings (summarizing the past 2 weeks before hospital admission) of eating disorder pathology, reported activity levels, exercise, and psychiatric symptoms

Variable	I felt—an increased urge to move around	Mentally restless	Physically restless
Eating Disorder Inventory‐2 (EDI‐2)			
Body dissatisfaction		.279*	
Ineffectiveness		.519**	
Perfectionism	.293*	.247*	
Interpersonal distrust			
Interoceptive awareness		.373**	.236*
Maturity fears		.453**	
Asceticism		.362**	
Impulse regulation		.420**	
Social insecurity		.301**	
Total score		.483**	
Beck Depression Inventory‐2 (BDI‐2)			
Total score		.341**	
Brief Symptom Inventory (BSI)			
Obsessive compulsion		.344**	
Interpersonal sensitivity		.335**	
Depression		.310*	
Anxiety	.254*	.532**	.454**
Hostility		.304*	
Phobic anxiety		.282*	
Psychoticism		.387**	
Global severity index		.428**	
Physically restless in the last 2 weeks		.404**	.534**
Daily routine exercise intensity in the last 2 weeks	.364**	.274*	
Compulsive exercise test (CET)			
Avoidance rule‐driven behavior	.338**	.348**	.305**
Weight control exercise	.278*	.293*	
Exercise for mood improvement	.297*	.308**	.351**
Lack of exercise enjoyment		.376**	
Exercise rigidity	.358**	.275*	.253*
Total score	.384**	.411**	.311**
Commitment to Exercise Scale (CES)			
CES_5 Do you continue to exercise at times when you feel tired or unwell.	.333**	.334**	.265*
Total score	.328**	.309**	.300*
Single Questionnaire Items			
BDI‐2_11 Agitation		.421**	.449**

* ≤.05, **≤.01.

## 4 DISCUSSION

In this study, over 80% of acutely hospitalized teenage patients with AN, all qualifying for the restricting subtype, reported they had experienced either, an increased urge to move, physical restlessness, or mental restlessness after significant weight loss. Two thirds of patients endorsed feeling alert, being able to concentrate and slightly fewer feeling motivated. Altogether, 95% of the AN patients reported experiencing at least one sign or a combination of two or all three signs.

The findings, based on the patients' reports and recollections, provide evidence that an increased urge to move, physical and mental restlessness exist as symptoms in AN and, if they can be confirmed in future studies, point to a disease‐specific weight loss accelerating metabolic adjustment in AN (Casper, 2018).

Remarkably, sensations of restlessness and the increased urge to move coexisted side by side with high levels of physical fatigue and low energy, and psychologically, with feeling tired and slowed down as well as with depressive symptoms and irritability. This suggests that just as persons subjected to simple starvation (Keys et al., 1950), AN patients perceive and suffer the full physiological and psychological effects of long‐lasting caloric deprivation. The high frequency of fatigue and loss of energy was unexpected, considering the patients' mobility and their usual silence concerning discomfort arising from prolonged undernutrition.

To our knowledge, no systematic studies on the prevalence of an increased urge to move, nor on physical restlessness and mental restlessness in restricting AN have been published. It is relevant to mention that the urge to move refers to spontaneous activity, that is, nonexercise activity thermogenesis (NEAT), as the energy expended for everything that is not sleeping, eating, or sports‐exercise (Levine, 2007), such as walking to school, typing, or fidgeting.

The Minnesota Experiments on Semistarvation (Keys et al., 1950) provide some information in healthy men, undernourished and underweight by 25%. Whereas the physical and psychological effects of semistarvation were similar to those observed here in AN, the results for what is called the “activity drive” and for physical restlessness differ substantially. The young men reported with increasing undernutrition a consistent decrease in the “activity drive” with the nadir at maximum weight loss. Physical restlessness was reported “sometimes” by 41% of the men, whereas depressive symptoms and irritability were endorsed by 66% and 62%, respectively, at the time of the lowest weight. On follow‐up, the men recalled experiencing lethargy, exhaustion and avoidance of exertion as prominent physical sensations associated with maximum weight loss (Eckert, Gottesman, Swigart, & Casper, 2018).

Together, the patients' answers on the RWLQ questionnaire capture an unusual picture of the motor response to severe weight loss, suggesting novel adjustment mechanisms to starvation in AN. As in simple starvation, the negative metabolic balance leads to increasing fatigue, tiredness, low energy, but instead of a consistent decline in the desire for movement, the continuing caloric deficit appears to trigger at some, as yet undetermined point, an increased urge to move as well as a high degree of physical restlessness in AN. These sensations coexist with and are counterbalanced by sensations typical of undernutrition, feeling physically and mentally tired, lacking energy and the wish to move less. The answers to the RWLQ questionnaire indicate that patients are aware of the existence of these mutually opposing sensations and are adapted to the experience. The sensations may contribute to the frequently observed compulsive exercise, a pathologically increased exercise pattern in eating disorders (Dittmer, Jacobi, & Voderholzer, 2018; Schlegl, Dittmer, Hoffmann, & Voderholzer, 2018).

It would be worth exploring how the sensations relate to a particular aspect of nonexercise activity, namely fidgeting. Work by Belak et al. (2017) found average activity (fidgeting) levels, while seated, to be 1.7 times greater in hospitalized AN patients than in healthy controls in recordings using an ingenuous novel SmartShoe device. Even low levels of fidgeting can increase energy expenditure above resting levels by 20%–40% (Levine, 2007, 2015). Interestingly, genetic factors have been found to contribute to the variance of nonexercise activity‐induced energy expenditure (Joosen, Gielen, Vlietinck, & Westerterp, 2005).

Through exploring the associations between the variables and with measures of eating disorder pathology and psychiatric comorbidity, it was possible to identify certain qualities of each of the principal variables. Importantly, the increased urge to move was related to the degree of weight loss, to feeling active, to movement despite feeling tired, to exercise intensity. The absence of correlations with the eating disorder or psychiatric pathology suggests that the increased urge to move might be an independent, likely physiological symptom.

Physical and mental restlessness were highly correlated with each other, with both related to weight loss. Recalled physical restlessness was related to actual physical restlessness, compulsion to exercise, anxiety and agitation experienced at the time of admission and to irritability and depressive feelings, emotional changes also accompanying semistarvation.

Physical restlessness in AN appears to differ from the motor restlessness in agitated depression with its depressive despair, by being associated with weight loss and a desire for exercise. This does not surprise, given that by its very nature, the psychomotor activation in agitated depression with its plaintive hand‐wringing, picking at objects and clothes and incessant purposeless wandering, along with expressions of intolerable anguish, derives from a different substrate, the affective bipolar spectrum (Akiskal, Benazzi, Perugi, & Rihmer, 2005).

Mental restlessness revealed itself as the most complex variable, strongly associated with distress of any kind, physical and mental fatigue, lack of concentration, with psychiatric symptoms, for example, with obsessionality, social insecurity, anxiety, psychoticism as well as with virtually all variables capturing eating disorder pathology, and signs on the compulsive exercise scale. Overall, mental restlessness appears to reflect most strongly the distress of the eating disorder, the psychological impairment and the totality of emotional suffering.

Our observations that a majority of patients reported being more active as children agree with the work of Davis et al. (1997) and further support their proposal for an essential role of physical activity in AN. Childhood activity levels were not related to the increased urge to move after weight loss. Variables in other studies, such as the “drive to exercise” (Keyes et al., 2015), composed of compulsive activity elements differ from the increased urge to move. The motor restlessness variable defined as “walking aimlessly on the ward” (Holtkamp et al., 2006) has similarities to the physical restlessness variable, indeed both were associated with the degree of starvation. By contrast, the “inner restlessness” variable, predefined as “anxious and jittery,” may be considered a component of the mental restlessness variable which had strong associations to eating disorder and psychiatric pathology.

### 4.1 Limitations

The use of a novel questionnaire is not only a strength, but also a limitation, as it highlights the need for replication and confirmation of the findings. Given the nature of the sensations, self‐report was required. The retrospective nature of the assessment is certainly a limitation, albeit it was by design, in that the inquiry into the sensations was focused around the time of the first maximum weight loss. Retrospective assessments, however, are liable to recall bias. By selecting young patients, we attempted to diminish the time to the recalled event. By naming the questionnaire “reactions to weight loss” and by listing the main variables in the fifth and seventh place we sought to reduce the likelihood of giving clues regarding the importance of different variables. On the other hand, retrospective recall tends to underestimate symptoms, raising the possibility that the findings in this study are underestimates. Also, it cannot be entirely ruled out that patients might not have misunderstood the increased urge for movement as “urge for increased movement,” that is, compulsive exercise.

## 5 CONCLUSIONS

The findings that AN patients experience an increased urge to move combined with physical and mental restlessness in response to prolonged caloric restriction during the development of AN fill an important gap in the chain of events producing spontaneous activity levels not different from normal‐weight controls. The increased urge to move and the physical restlessness likely triggered by as yet unknown starvation‐related processes suggest a unique, dysfunctional physiological adaptation of energy regulating pathways in AN (Casper, 2016). Future studies might explore whether without these unique adaptations, continued activity in AN would be possible in the presence of concurrent physical and mental fatigue.

## CONFLICT OF INTEREST

The authors have no conflict of interest.
