# Supplementary material for: Increased urge for movement, physical and mental restlessness, fundamental symptoms of restricting anorexia nervosa?
Source: Brain Behav. 2020 Feb 4;10(3):e01556. doi: 10.1002/brb3.1556 (PMC7066368; doi:10.1002/brb3.1556)
Supplement: Supplementary file 1 [file BRB3-10-e01556-s001.docx]

**Appendix 1**

**Reactions to Weight Loss Questionnaire (RWLQ)**

**Dear Patient,**

We would like to understand better how different people react to changes in their body weight.

We would appreciate it, if you could help us by answering the questionnaire/questions below.

Date:__________ Age:___________ Height:__________ Actual weight :___________lbs

Lowest weight:_______lbs; when________Month/Year;

highest weight:_______; when_____ Month/Year

**Looking back:**

When I considerably lost weight for the first time in my life (from______ lbs to_____ lbs

= ____ lbs in _____ months, this was approximately _________month ______year

I felt .........

**Physically**

|  | ***Please check*** | | |
| --- | --- | --- | --- |
|  | **True** | **Partially true** | **Not true** |
| active |  |  |  |
| full of energy |  |  |  |
| tired |  |  |  |
| having no energy |  |  |  |
| I felt an increased desire to move around |  |  |  |
| I preferred to move less |  |  |  |
| restless |  |  |  |
| with as much energy as I felt before I lost weight |  |  |  |

**Mentally**

|  | ***Please check*** | | |
| --- | --- | --- | --- |
|  | **True** | **Partially true** | **Not true** |
| motivated |  |  |  |
| full of energy |  |  |  |
| tired |  |  |  |
| no energy |  |  |  |
| restless |  |  |  |
| unmotivated |  |  |  |
| slowed down |  |  |  |
| I could concentrate |  |  |  |
| I was alert |  |  |  |
| depressed |  |  |  |
| Irritable |  |  |  |

How well do you remember the times when you had considerably lost of weight for the first time?

□ very well □ well □ weakly □ poorly

When I was a child I moved around more than other children. Please check

□ true □ partially true □ not true

**Thank you very much for your help**
